# The Interplay between ESIPT and TADF for the 2,2′-Bipyridine-3,3′-diol: A Theoretical Reconsideration

**DOI:** 10.3390/ijms232213969

**Published:** 2022-11-12

**Authors:** Xin Zhao, Lixia Zhu, Qi Li, Hang Yin, Ying Shi

**Affiliations:** Institute of Atomic and Molecular Physics, Jilin University, Changchun 130012, China

**Keywords:** excited-state intramolecular proton transfer, thermally activated delayed fluorescence, spin-orbit coupling, hydrogen-bond dynamics, electron-hole distribution

## Abstract

Organic molecules with excited-state intramolecular proton transfer (ESIPT) and thermally activated delayed fluorescence (TADF) properties have great potential for realizing efficient organic light-emitting diodes (OLEDs). Furthermore, 2,2′-bipyridine-3,3′-diol (BP(OH)_2_) is a typical molecule with ESIPT and TADF properties. Previously, the double ESIPT state was proved to be a luminescent state, and the T_2_ state plays a dominant role in TADF for the molecule. Nevertheless, whether BP(OH)_2_ undergoes a double or single ESIPT process is controversial. Since different ESIPT channels will bring different TADF mechanisms, the previously proposed TADF mechanism based on the double ESIPT structure for BP(OH)_2_ needs to be reconsidered. Herein, reduced density gradient, potential energy surface, IR spectra and exited-state hydrogen-bond dynamics computations confirm that BP(OH)_2_ undergoes the barrierless single ESIPT process rather than the double ESIPT process with a barrier. Moreover, based on the single ESIPT structure, we calculated spin-orbit coupling matrix elements, nonradiative rates and electron-hole distributions. These results disclose that the T_3_ state plays a predominant role in TADF. Our investigation provides a better understanding on the TADF mechanism in hydrogen-bonded molecular systems and the interaction between ESIPT and TADF, which further provides a reference for developing efficient OLEDs.

## 1. Introduction

Organic molecules with thermally activated delayed fluorescence (TADF) properties have aroused extensive attention due to their significant applications in organic light-emitting diodes (OLEDs) [1,2,3,4,5]. These applications are attributed to their high utilization of both singlet and triplet excitons. For conventional fluorescent OLEDs, only 25% singlet excitons are available, which causes low internal quantum efficiency [6,7]. In contrast, despite the fact that phosphorescent OLEDs can take full advantage of excitons by doping iridium or platinum complexes, their presence brings high costs and environmental pollution [8,9]. In 2012, Adachi’s team realized 100% internal quantum efficiency using TADF OLEDs [10]. TADF is the phenomenon of converting triplet excitons to singlet states via a fast RISC process under thermal activation [11,12]. Indeed, the luminous efficiency of molecules with the TADF properties suffer from the self-absorption effect, which hampers the efficiency improvement and affects practical application [13]. It is worth noting that the organic molecules with excited-state intramolecular proton transfer (ESIPT) properties can avoid this problem due to the significant Stokes shift [14,15,16,17,18]. ESIPT can be regarded as an ultrafast photo-tautomerization process in which a proton is transferred from proton donor to proton acceptor along with a strong intramolecular hydrogen bond (IHB) [19,20,21,22,23]. Therefore, the research of the organic TADF molecules capable of ESIPT effectively facilitates internal quantum efficiency, characterized by high luminous efficiency, low cost and long lifetime [24,25].

Additionally, 2,2′-bipyridine-3,3′-diol (BP(OH)_2_) is a typical molecule that simultaneously exhibits the ESIPT properties and TADF features. Bulska et al. reported that BP(OH)_2_ under the S_1_ state would undergo a cooperative double ESIPT reaction [26]. Based on the double ESIPT form, Tokumura et al. put forward the TADF mechanism for BP(OH)_2_ in hexane [27]. Concretely, after photoexcitation, the rapid double ESIPT happens, and then the T_2_ structure can be obtained through the intersystem crossing (ISC) process in competition with emissions from the S_1_ state. Following the ISC process, the reverse intersystem crossing (RISC) process from T_2_ to S_1_ state occurs, which thereby results in the TADF phenomenon. It was found that only the S_1_ and T_2_ states participate in the ISC and RISC processes. Plasser et al. deemed that BP(OH)_2_ prefers to occur in the stepwise double ESIPT process rather than in the concerted double ESIPT process, based on the TDDFT and resolution-of-identity second-order approximate coupled-cluster methods [28]. Recently, Zhao et al. suggested that, in low-polar solvents, the single ESIPT is more likely to occur in BP(OH)_2_ [29]_._ Accordingly, whether the molecule undergoes single or double proton transfer has not been identified completely. However, different geometric structures of the S_1_ state significantly affect the spin-orbit coupling interaction between the singlet and triplet states, which further brings different TADF mechanisms [30,31]. Therefore, the previously proposed TADF mechanism based on the double ESIPT structure needs to be reconsidered.

In this study, we have demonstrated the ESIPT pathway and reconsidered the TADF mechanism of BP(OH)_2_ theoretically in low-polar hexane solvent on account of density functional theory (DFT) and time-dependent density functional theory (TDDFT) methods. Specifically, the optimized geometric structures and absorption and emission peaks were analyzed to investigate the excited-state behavior for BP(OH)_2_. In addition, the calculations of the frontier molecular orbitals (FMOs), infrared (IR) spectra, reduced density gradient (RDG), potential energy curves (PECs), potential energy surface (PES), and excited-state hydrogen-bond dynamics were applied to explore the ESIPT pathway. Moreover, the spin-orbit coupling (SOC) constants, energy level gaps and nonradiative decay rates were calculated to describe the TADF mechanism of BP(OH)_2_. Furthermore, the electron-hole distribution was applied to analyze the electron excitation nature. In a word, our theoretical investigations demonstrate that BP(OH)_2_ will undergo the single ESIPT process rather than the double ESIPT process, and provide reconsiderations of the mechanisms of the ISC and RISC processes involved in the TADF phenomenon of this molecule. These findings provide a theoretical foundation for understanding how the ESIPT mechanism affects TADF, which favors achieving highly efficient photoluminescence.

## 2. Results and Discussion

### 2.1. Structural Analysis and Absorption and Fluorescence Spectra

**Figure 1:**
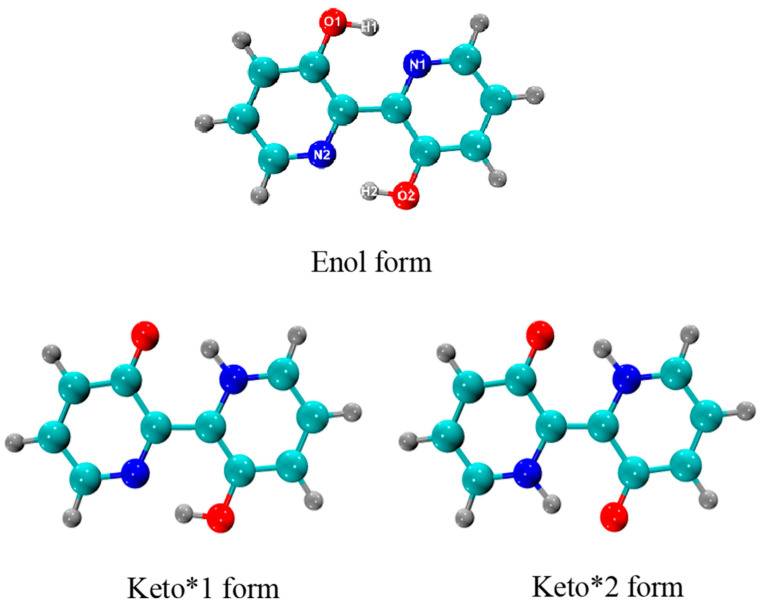
The optimized structure of BP(OH)_2_ in the S_0_ and S_1_ states at the B3LYP/def2TZVP (gd3bj) IEFPCM (Hexane) level. Enol form: the initial conformation at the S_0_ state; Keto*1 form: the conformation based on single ESIPT in the S_1_ state; Keto*2 form: the conformation based on double ESIPT in the S_1_ state. The blue atom represents N, the cyan atom represents C, the grey atom represents H, and red atom represents O.

Figure 1 presents optimized geometric structures of S_0_ and S_1_ states for BP(OH)_2_ in hexane at the B3LYP/def2-TZVP level with the dispersion correction (gd3bj). Meanwhile, the primary structural parameters involved in the IHBs of BP(OH)_2_ in hexane are collected in Table 1. The optimized configurations show that the Enol* form for BP(OH)_2_ does not exist in the S_1_ state. In addition, there are two possible ESIPT pathways in the S_1_ state: the single ESIPT path and double ESIPT path. For the single proton transfer path, the distance between N1 and H1 decreases from 1.659 Å to 1.026 Å, and that between O1 and H1 is elongated from 1.005 Å to 1.788 Å. For the double proton transfer path, the N1-H1 length is shortened from 1.659 Å to 1.034 Å, and O1-H1 is stretched from 1.005 Å to 1.725 Å. Similar to N1-H1 and O1-H1, the N2-H2 distance is decreased, while the length of O2–H2 exhibits a tendency of elongation. Therefore, the ESIPT pathways for BP(OH)_2_ can be either the single ESIPT path or the double ESIPT path.

The absorption and fluorescence peaks for BP(OH)_2_ in hexane were calculated at the B3LYP/def2-TZVP (gd3bj) level. The relevant data are recorded in Table 2. The theoretical absorption maximum of the Enol form locates at 337 nm, being consistent with the experimental data (345 nm) [27]. This illustrates the dependability of our chosen calculation method for BP(OH)_2_ system. The fluorescence peaks emitted from the optimized Keto*1 and Keto*2 forms are found at 484 nm and 479 nm, respectively, which coincide with the experimental peak (499 nm). Thus, the fluorescence is believed to be derived from the Keto*1 and Keto*2 structures combined with the optimized structures. However, in Table 2, the fluorescence peak of the Keto*1 conformation is in better agreement with 499 nm than that of the Keto*2 conformation.

### 2.2. Frontier Molecular Orbitals

**Figure 2:**
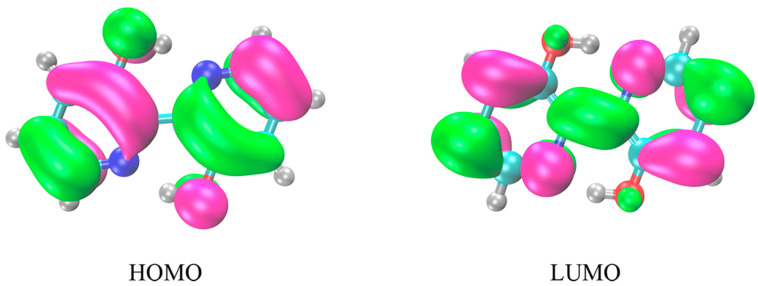
The HOMO and LUMO of BP(OH)_2_ in hexane solvent.

The tendency of the ESIPT behavior can be effectively affected by charge distribution changes [32]. For exploring the mechanism of the ESIPT process, we analyzed the charge distribution for the FMOs (HOMO and LUMO) of the S_0_ and S_1_ states in BP(OH)_2_, as drawn in Figure 2. We find that the transition from HOMO to LUMO is corresponding to a dominant π-π∗ type character, which plays a significant role in promoting the proton transfer. Herein, we focus only on the charge distribution around the IHB, affecting the excited-state hydrogen bond dynamics. As detailed in Figure 2, the electron density distribution of both hydroxyl oxygens, which are related to the ESIPT process, decreases, whereas that of the proton acceptors increases from HOMO to LUMO. These changes mean that the electronegativities of the nitrogen atoms are stronger than those of the oxygen atoms for BP(OH)_2_ after the transition from HOMO to LUMO. Therefore, the FMOs analysis illustrates that both IHBs in BP(OH)_2_ are reinforced upon excitation to S_1_, which in turn facilitates the occurrence of the proton transfer process.

### 2.3. Reduced Density Gradient Analysis

RDG can clearly characterize the interactions of molecules in real space and the intensity of the interactions [15,33]. Specifically, RDG can be expressed as the following Formula (1):

(1) RDG(r)=12(3π2)13|∇ρ(r)|ρ(r)43

In addition, the electron density ρ(r) is associated with the eigenvalues I_2_ in the Hessian matrix of electron density, as shown in Equation (2)

(2) $$\Omega \left(r\right)=\mathrm{sign}\left(I_{2}\left(r\right)\right)\rho \left(r\right)$$

Herein, the Ω(r) <0 exhibits hydrogen-bonding interactions. The Ω(r) ≈0 stands for the van der Waals interaction. The Ω(r) >0 refers to the steric effect.

**Figure 3:**
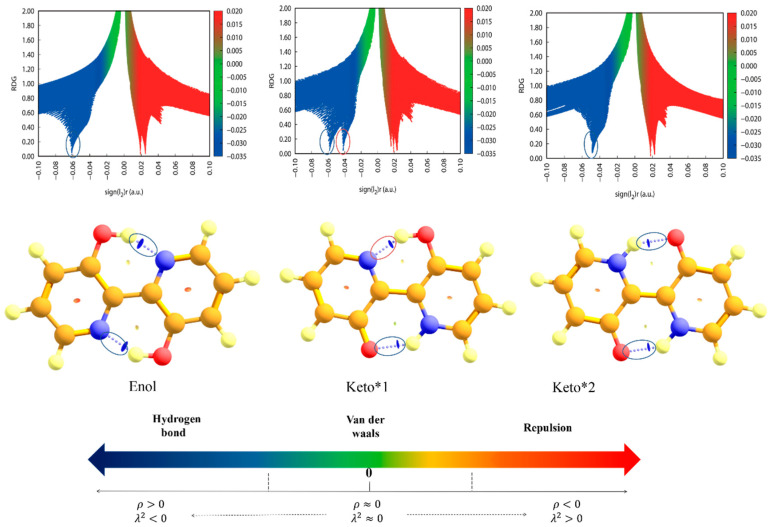
Diagrams of RDG (Function 1) vs. Ω(r) (Function 2) and gradient isosurfaces of BP(OH)_2_ at Enol, Keto*1 and Keto*2 states.

As for BP(OH)_2_, to unveil the location and strength of IHB interactions and further judge the ESIPT pathway, the RDG image and gradient isosurfaces of this molecule are provided in Figure 3. The spikes inside the circles denote the strength of the hydrogen bond. Additionally, there is a blue disk between the oxygen and hydrogen atoms, and the hydrogen and nitrogen atoms, corresponding to the spikes in the circles. As detailed in Figure 3, the spike position around −0.06 for BP(OH)_2_ reveals the hydrogen-bonding interactions of O2-H2…N2 and O1-H1…N1 at the S_0_ state. For the Keto*1 state, the two spike peaks located around −0.06 and −0.04 are attributed to O1-H1…N1 and O2…H2-N2, respectively. The IHBs corresponding to the Keto*2 state are O2…H2-N2 and O1…H1-N1 with the spike peak of −0.05. During the process from the S_0_ to Keto*1 state, the IHB changes; namely, the proton H1 is transferred to N1 atoms. Moreover, the peak position of O2-H2…N2 shifted from −0.06 of the S_0_ state to −0.04 of the Keto*1 state, implying that the intensity of IHB (O2-H2…N2) is weakened. The change of two IHBs means that only single ESIPT reaction can occur in BP(OH)_2_. During the process from the S_0_ to the Keto*2 state, the hydrogen bond positions change from O1-H1…N1 and O2 H2…N2 to O1…H1-N1 and O2…H2-N2. However, the absolute spike peaks (0.04) of the Keto*2 state are smaller than that of the O1…H1-N1 in the Keto*1 state (0.05). This indicates that the strength of the newly formed IHB after the single ESIPT process is significantly stronger than those of the two IHBs after the double ESIPT process. Therefore, these results confirm that the intramolecular single ESIPT reaction is more likely to occur than the double ESIPT reaction.

### 2.4. Potential Energy Surface and Potential Energy Curves

**Figure 4:**
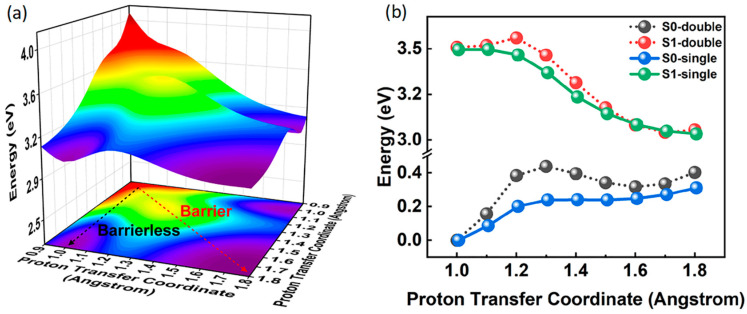
Constructed (**a**) PES in the S_1_ state and (**b**) PECs in the S_0_ and S_1_ states for BP(OH)_2_.

In order to determine whether BP(OH)_2_ takes place in the single or double proton transfer process, we constructed the PES of the S_1_ state as a function of the bond lengths of O1-H1 and O2-H2. The PES is drawn in Figure 4a, where the black and red arrows represent the single ESIPT pathway and double ESIPT pathway, respectively. Importantly, the four stable points can be observed in Figure 4a. The PES of the S_1_ state is symmetrical along the diagonal curve as a result of the symmetrical structure of BP(OH)_2_. This means that BP(OH)_2_ has three stable conformations in the S_1_ state: the Enol* form, the Keto*1 form and the Keto*2 form. Specifically, in order to achieve the stable Keto*2 form, the double ESIPT pathway needs to surpass an energy barrier from the Enol* form to the Keto*2 form. However, there is no potential barrier in the single ESIPT pathway along with O1-H1…N1 or O2-H2…N2. That is to say, the single ESIPT process will happen spontaneously when forming the Keto*1 conformation upon excitation to S_1_. Consequently, the BP(OH)_2_ in hexane will occur during the single ESIPT process instead of the double ESIPT, even though it possesses two IHBs.

We further plotted the PECs in Figure 4b to compare the difference between the double and single proton transfer pathways clearly. The S_0_ single curve shows that BP(OH)_2_ exists in the Enol configuration in the S_0_ state. For the S_0_ double path, the high energy barrier prevents the double proton transfer behavior from taking place. These results mean that neither the single nor double proton transfer process can occur in the S_0_ state. In addition, we notice that, for the S_1_ double path, BP(OH)_2_ needs to undergo an energy barrier forming two new hydrogen bonds (N1-H1 and N2-H2), while for the S_1_ single path, there is only one stable structure, and the coordinate of this stable configuration is 1.724 Å. In other words, the BP(OH)_2_ will take place in a barrierless single proton transfer process. Thus, it can be further attested that the double ESIPT pathway is ruled out due to the existence of a potential barrier.

### 2.5. Infrared Spectra

**Figure 5:**
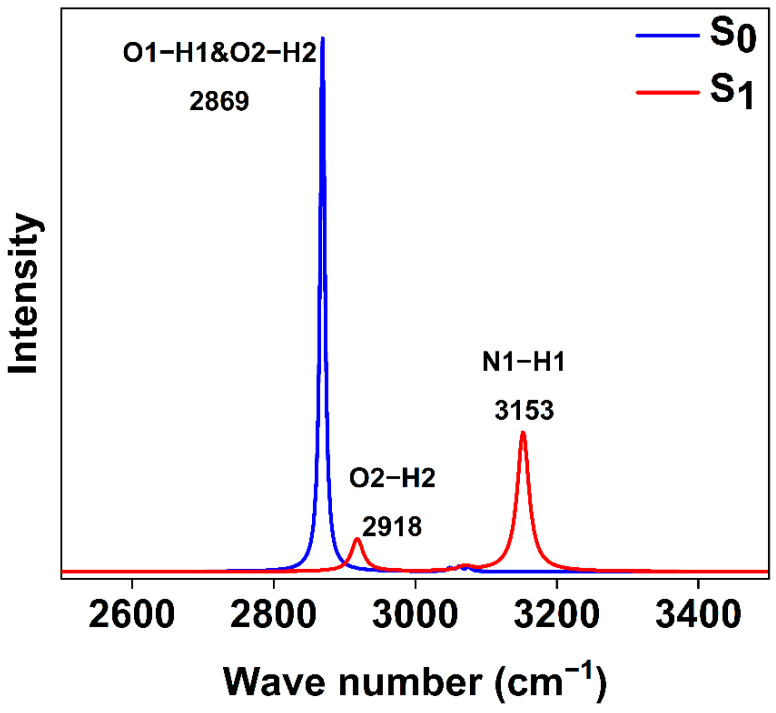
Calculated S_0_ state and S_1_ state infrared spectrum based on the single ESIPT structure of BP(OH)_2_ in hexane.

IR spectra are found to be an invaluable tool to describe hydrogen-bonding dynamics processes by analyzing the changes in vibration frequency [34]. To affirm our viewpoint once again—that only the single ESIPT occurs in the S_1_ state—Figure 5 presents IR spectra of BP(OH)_2_ (scaling factor 0.959) in the S_0_ and S_1_ states. The calculated IR spectra range from 2600 cm^−1^ to 3400 cm^−1^, which involve vibration peaks for O1-H1, O2-H2 and N1-H1 groups. Specifically, in the S_0_ state, there is only a characteristic peak around 2869 cm^−1^ due to the structural symmetry of BP(OH)_2_, assigned to the O1-H1 and O2-H2 stretching vibration. When excited to S_1_, the O2-H2 and N1-H1 stretching vibrational frequency of BP(OH)_2_ are at 2918 cm^−1^ and 3153 cm^−1^, respectively. Obviously, the IR spectrum of the S_1_ state for BP(OH)_2_ is different from that of the S_0_ state. The vibration peak of O2-H2 exhibits a blue shift from 2869 cm^−1^ of the S_0_ state to 2918 cm^−1^ of the S_1_ state. This reveals that, following excitation to S_1_, the IHB (O2-H2…N2) is weakened, which is not conducive to the transfer of H2 protons. Interestingly, the O1-H1 vibration peak (2869 cm^−1^) disappears in the S_1_ state while the new N1-H1 peak (3153 cm^−1^) forms, indicating the transfer of proton H1 from O1 to N1. Consequently, these results above demonstrate the occurrence of the single proton transfer process upon excitation.

### 2.6. Excited State Hydrogen Bond Dynamics

**Figure 6:**
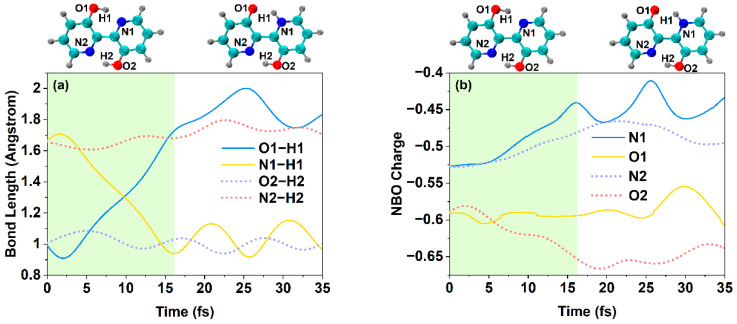
(**a**) The important bond lengths with time evolution, including the Enol* form (left) and Keto*1 form (right) and (**b**) the NBO charge of primary atoms with time evolution for BP(OH)_2_, including the Enol* form (left) and Keto*1 form (right). Atoms of different colors in the Enol* and Keto* are represented as in Figure 1.

Considering that the timescale and characteristics of the ESIPT process may well be theoretically simulated and further revealed by excited-state hydrogen-bonding dynamics [35], the time-evolution trajectories of all bond lengths involved in IHBs are presented in Figure 6a. The Enol* form is the initial photoexcited configuration. In addition, at ~15 fs, the bond length of O1-H1 increases gradually with the time evolution, and the H1-N1 decreases gradually with the time evolution in comparison with the bond lengths of the Enol* form. These changes are due to the fact that the proton H1 moves along with a hydrogen bond from O1 to N1 rapidly until the proton H1 is transferred. After 15 fs, all the bond lengths related to the intramolecular hydrogen bond tend toward dynamic equilibrium. The Keto*1 form after 15 fs can be seen in Figure 6a. By comparing the Enol* form with the Keto*1 form, only the single ESIPT takes place in BP(OH)_2_ upon excitation to the S_1_ state.

Due to the intramolecular charge redistribution having momentous effects on the ESIPT behavior, we also calculated the time evolution of the NBO charge for the atoms related to the proton transfer displayed in Figure 6b. For O1-H1…N1, within 15 fs, the negative charge of the N1 atom decreases, while the electronegativity of O1 hardly obviously changes with time evolution. This phenomenon is due to the fact that the positively charged proton H1 gradually moves away from O1 to the N1 atom, which offsets the negative charge on N1. At the same time, the electronegativity of O2 increases gradually with time evolution, but the negative charge of N2 decreases with time evolution. These changes mean that the interaction between O2 and H2 is enhanced and the interaction between N2 and H2 atoms is weakened within 15 fs. Thus, it is not possible to transfer the proton H2 from O2 to N2. The NBO charge of all atoms related to the intramolecular hydrogen bond tends toward dynamic equilibrium after 15 fs. Based on the above analysis, the variety of the NBO charge once again supports the viewpoint that the BP(OH)_2_ molecule will carry out the single proton transfer reaction instead of the double proton transfer upon excitation.

### 2.7. Spin-Orbit Coupling Interaction

The BP(OH)_2_ molecule was found to exhibit not only single proton transfer properties but also the TADF phenomenon [27]. TADF phenomenon is closely correlated with the ISC and RISC processes. According to Fermi’s golden rule [36], the larger SOC constant and the smaller energy level gap can promote the ISC and RISC processes. Therefore, to verify which triplet states are involved in the ISC process and elucidate the mechanism of the delayed fluorescence phenomenon, the energy level gaps and SOC constants are recorded in Table 3. To be specific, the energy level gaps for S_1_-T_1_, S_1_-T_2_ and S_1_-T_3_ are 0.980 eV, 0.120 eV and −0.046 eV, respectively. The energy level gaps of S_1_-T_3_ and S_1_-T_2_ are smaller than that of S_1_-T_1_, which makes the T_1_ state difficult to induce the RISC process from T_1_ to S_1_. Based on that, the SOC_ISC_ constants of S_1_-T_3_ and S_1_-T_2_ are 31.029 cm^−1^ and 0.411 cm^−1^. Obviously, the SOC_ISC_ constant of S_1_-T_3_ is much larger than that of S_1_-T_2_. Thus, the energy level gap, combined with spin-orbit coupling interaction, confirms that the ISC process is mainly the transition from the S_1_ to the T_3_ state. Correspondingly, it can be found that the SOC_RISC_ constants of S_1_-T_1_, S_1_-T_2_ and S_1_-T_3_ are 0.095 cm^−1^, 0.585 cm^−1^ and 0.321 cm^−1^, respectively. The SOC_RISC_ constants of S_1_-T_3_ and S_1_-T_2_ are larger than those of T_1_-S_1_, implying that the RISC process of T_2_-S_1_ and T_3_-S_1_ would be realized facilely.

**Figure 7:**
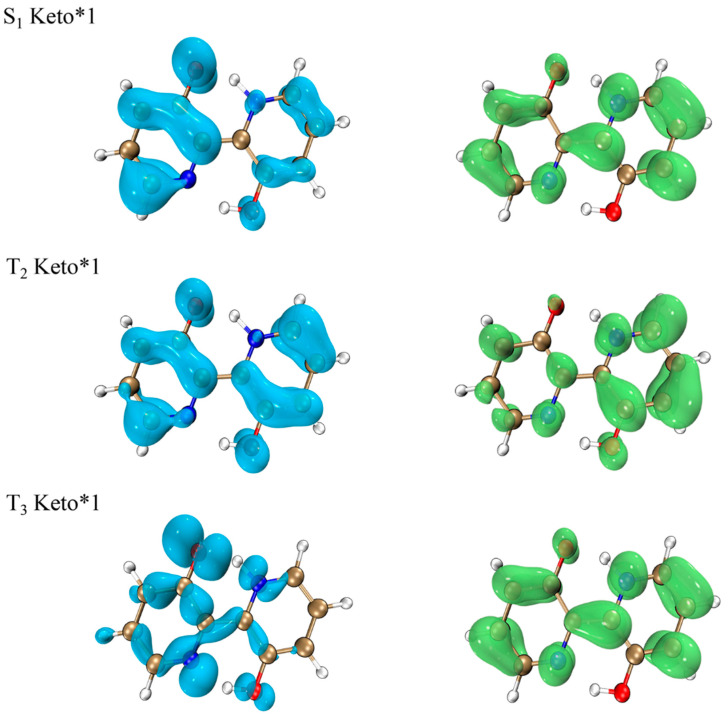
Electron-hole distribution based on the Keto*1 form in the excited states for BP(OH)_2_. Blue color and green color represent holes and electrons, respectively. The golden atom represents C, the blue atom represents N, the red atom represents O, and light grey atom represents H.

Since differences in the electron-hole distribution between the singlet and triplet states will affect the ISC process [37], we calculated the electron-hole distribution diagrams of the singlet (S_1_ Keto*1) and triplet (T_2_ Keto*1 and T_3_ Keto*1) states, as plotted in Figure 7. In accordance with the El-Sayed rule [38], the transition between identical vibrational states is forbidden. Although the S_1_, T_2_ and T_3_ states are characteristic of charge transfer, the electron-hole distribution of the S_1_ state is different from that of T_2_ and T_3_ states. This means that there are two ISC transition channels, which are from the S_1_ state to T_2_ and from the S_1_ state to T_3_. During the excitation process, the T_3_ state undergoes significant electron transfer and drastic alternation of electron distribution structure. In other words, the electron transfer from the S_1_ state to T_3_ is more pronounced than that from the S_1_ state to T_2_, which is the reason for the large SOC_ISC_ value between the S_1_ and T_3_ states. However, the degree of electron transfer in the S_1_ state is close to that in the T_2_ state, leading to a smaller SOC_ISC_ value compared with SOC_ISC_ of the S_1_ and T_3_ states. Meanwhile, we find from Table 3 that the T_2_ energy level is rather close to the T_3_ energy level, which means that there may be an internal conversion process from the T_3_ state to T_2_. Thus, it is demonstrated that the T_3_ energy level plays a significant role in collecting triplet state excitons.

For a more in-depth discussion of the ISC and RISC processes associated with TADF, the ISC and RISC rates of BP(OH)_2_ in hexane are listed in Table 3. The ISC rates of S_1_-T_1_, S_1_-T_2_ and S_1_-T_3_ were calculated to be 1.40 × 10^6^ s^−1^, 1.73 × 10^6^ s^−1^ and 1.05 × 10^10^ s^−1^, respectively. The ISC rate of S_1_-T_3_ is four orders of magnitude larger than that of S_1_-T_1_ and S_1_-T_2_ due to the small energy level gap and the large SOC constant. Consequently, the result demonstrates that the ISC process will primarily happen from the S_1_ state to T_3_. Furthermore, the RISC rates of S_1_-T_2_ and S_1_-T_3_ are 3.52 × 10^6^ s^−1^ and 1.12 × 10^6^ s^−1^, which are one order of magnitude larger than that of S_1_-T_1_. Thus, it is confirmed once again that the RISC process belongs to the transition from the T_3_ state to S_1_ and the one from the T_2_ state to S_1_. Through the above analysis, our calculated results illustrate that the ISC process is mainly assigned to the transition from the S_1_ state to T_3_, and the RISC process in BP(OH)_2_ belongs to the transition from the T_3_ state to S_1_ and the one from the T_2_ state to S_1_. In the previous paper of Tokumura et al. [27], for BP(OH)_2_, the ISC and RISC processes after the double proton transfer are the transition between the S_1_ and T_2_ states. In contrast, herein, we reconsider the two processes based on the single proton transfer structure (Keto*1 form).

### 2.8. TADF Mechanism

**Figure 8:**
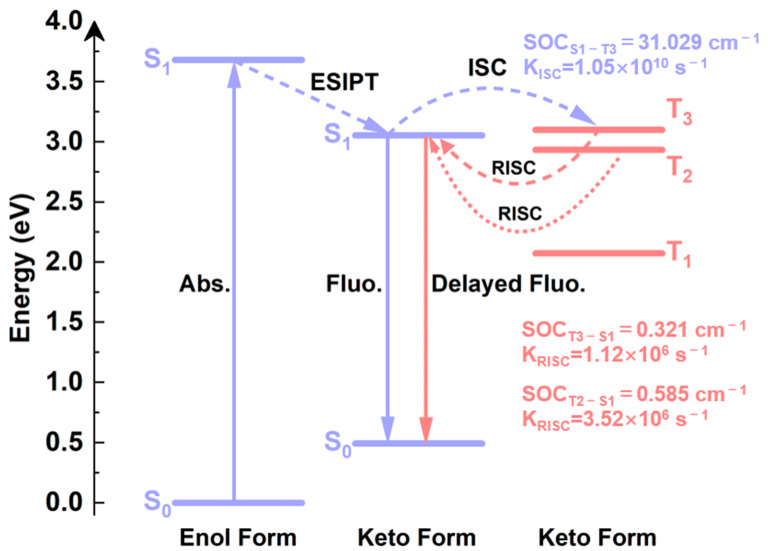
The schematic diagram of photoluminescence processes for BP(OH)_2_ in hexane solvent.

Based on the analysis above, we describe the photophysical processes that give rise to the TADF phenomenon in BP(OH)_2_, with the ESIPT feature displayed in Figure 8. Upon excitation to the S_1_ state, BP(OH)_2_ occurs in the intramolecular single proton transfer, forming the S_1_ Keto*1 structure. Subsequently, the normal emission from the S_1_ Keto*1 conformation occurs in competition with the efficient ISC process from the S_1_ Keto*1 state to T_3_ Keto*1. Following the ISC process, there are two possible RISC pathways: the transition from the T_3_ Keto*1 state to S_1_ Keto*1 and the one from the T_2_ Keto*1 state to S_1_ Keto*1. The BP(OH)_2_ molecule that returns to the S_1_ Keto*1 state decays to the S_0_ Keto state by emitting delayed fluorescence. In summary, the luminescence is derived from the single ESIPT structure, and the T_3_ state plays a crucial role in the TADF process due to the large electron-hole separation.

## 3. Materials and Methods

All calculations in our work were completed using the DFT and TDDFT methods at the B3LYP/def2-TZVP level with the dispersion correction (gd3bj) [39,40,41,42,43,44]. The effect of the hexane solvent was considered with the integral equation formalism variant of the polarizable continuum model [45]. In this way, the optimized geometric structures, absorption and emission properties, FMOs, IR spectra, PECs, PES and excited-state hydrogen-bond dynamics were calculated in the Gaussian 16 package [46]. Vibrational frequencies were computed by determining the second derivatives of the energy with respect to the Cartesian nuclear coordinates and then transforming to mass-weighted coordinates. Concerning the ab initio molecular dynamics study of ESIPT, the classical trajectory calculation (i.e., Born–Oppenheimer molecular dynamics (BOMD)) was undertaken in the S_1_ state [47,48]. The RDG image and the electron-hole distribution of the singlet and triplet states were analyzed using Multiwfn software [49]. In addition, the PySOC program interfaced with Gaussian 16 software was used to calculate the SOC constants [50,51]. Moreover, the nonradiative decay rates between singlet and triplet states were investigated in MOMAP [52,53,54,55].

## 4. Conclusions

The ESIPT-based TADF mechanisms of BP(OH)_2_ were theoretically reconsidered at the B3LYP/def-2TZVP (gd3bj) level. Geometric structure, RDG scatter plots, PES and IR spectra indicate that BP(OH)_2_ in hexane will undergo the single ESIPT process rather than the double ESIPT upon excitation to S_1_ state. Furthermore, the BOMD computations prove once again the occurrence of the single ESIPT process and further provide the timescale for this process. Moreover, the energy level gaps, SOC constants and nonradiative decay rates of S_1_-T_2_, S_1_-T_2_ and S_1_-T_2_ were calculated based on the single ESIPT structure (Keto*1 form). These results elucidate that the ISC process is mainly the transition from the S_1_ Keto*1 state to T_3_ Keto*1, and the RISC process belongs to the transition from the T_3_ Keto*1 state to S_1_ Keto*1 and the one from the T_2_ Keto*1 state to S_1_ Keto*1. Furthermore, we find that the different electron-hole distributions between the singlet and triplet states of BP(OH)_2_ facilitate the ISC process utilizing the electron-hole analysis. Thus, the T_3_ state plays a dominant role in the TADF process, which is induced by the significant electron-hole separation between S_1_ and T_3_ states. Our work will not only be helpful to further understand the interaction between ESIPT and TADF but also contribute to achieving high luminescence efficiency for organic molecules with TADF properties.

## Figures and Tables

**Table 1:** Obtained primary bond lengths for the optimized structure of BP(OH)_2_ at the S_0_ and S_1_ states (Unit Å).

Unit Å	Enol	Keto*1	Keto*2
N1-H1 (Å)	1.659	1.026	1.034
O1-H1 (Å)	1.005	1.788	1.725
N2-H2 (Å)	1.659	1.686	1.034
O2-H2 (Å)	1.000	1.000	1.724

**Table 2:** The absorption and emission peaks of BP(OH)_2_ in hexane solvent.

	Abs. (nm)	Fluo. (nm)	
Exp.	345	499	
Theor.	337(Enol)	484(Keto*1)	479(Keto*2)

**Table 3:** Calculated SOC constants, energy level difference and rates of ISC (K_ISC_) and RISC (K_RISC_) between the singlet and triplet states of BP(OH)_2_ in hexane.

	S_1_-T_1_	S_1_-T_2_	S_1_-T_3_
ΔE (eV)	0.980	0.120	−0.046
SOC_ISC_ (cm^−1^)	0.152	0.411	31.029
SOC_RISC_ (cm^−1^)	0.095	0.585	0.321
K_ISC_ (s^−1^)	1.40 × 10^6^	1.73 × 10^6^	1.05 × 10^10^
K_RISC_ (s^−1^)	5.48 × 10^5^	3.52 × 10^6^	1.12 × 10^6^

## Data Availability

Not applicable.
